# Responding to a surge in overdose deaths: perspectives from US syringe services programs

**DOI:** 10.1186/s12954-022-00664-y

**Published:** 2022-07-19

**Authors:** Madeline C. Frost, Elizabeth J. Austin, Maria A. Corcorran, Elsa S. Briggs, Czarina N. Behrends, Alexa M. Juarez, Noah D. Frank, Elise Healy, Stephanie M. Prohaska, Paul A. LaKosky, Shashi N. Kapadia, David C. Perlman, Bruce R. Schackman, Don C. Des Jarlais, Emily C. Williams, Sara N. Glick

**Affiliations:** 1 Department of Health Systems and Population Health, School of Public Health, University of Washington, 1959 NE Pacific St, Seattle, WA 98195 USA; 2 Health Services Research and Development Center of Innovation for Veteran-Centered and Value-Driven Care, Veterans Affairs Puget Sound Health Care System, 1660 S Columbian Way, Seattle, WA 98108 USA; 3 Division of Allergy and Infectious Diseases, Department of Medicine, University of Washington, 1959 NE Pacific St, Seattle, WA 98195 USA; 4 Department of Population Health Sciences, Weill Cornell Medical College, 418 E 71st St #21, New York, NY 10021 USA; 5 Dave Purchase Project, North American Syringe Exchange Network, 535 Dock Street Suite 113, Tacoma, WA 98402 USA; 6 Division of Infectious Diseases, Weill Cornell Medical College, 418 E 71st St #21, New York, NY 10021 USA; 7 Division of Infectious Diseases, Department of Medicine, Icahn School of Medicine at Mount Sinai, 1 Gustave L. Levy Pl, New York, NY 10029 USA; 8 Center for Drug Use and HIV/HCV Research, 708 Broadway, 4th Floor, New York, NY 10003 USA; 9 School of Global Public Health, New York University, 708 Broadway, New York, NY 10003 USA

**Keywords:** Syringe services program, Syringe exchange, Opioids, Fentanyl, Overdose, Harm reduction, Naloxone, COVID-19, Coronavirus, Qualitative

## Abstract

**Background:**

US overdose deaths have reached a record high. Syringe services programs (SSPs) play a critical role in addressing this crisis by providing multiple services to people who use drugs (PWUD) that help prevent overdose death. This study examined the perspectives of leadership and staff from a geographically diverse sample of US SSPs on factors contributing to the overdose surge, their organization’s response, and ongoing barriers to preventing overdose death.

**Methods:**

From 2/11/2021 to 4/23/2021, we conducted semi-structured interviews with leadership and staff from 27 SSPs sampled from the North American Syringe Exchange Network directory. Interviews were transcribed and qualitatively analyzed using a Rapid Assessment Process.

**Results:**

Respondents reported that increased intentional and unintentional fentanyl use (both alone and combined with other substances) was a major driver of the overdose surge. They also described how the COVID-19 pandemic increased solitary drug use and led to abrupt increases in use due to life disruptions and worsened mental health among PWUD. In response to this surge, SSPs have increased naloxone distribution, including providing more doses per person and expanding distribution to people using non-opioid drugs. They are also adapting overdose prevention education to increase awareness of fentanyl risks, including for people using non-opioid drugs. Some are distributing fentanyl test strips, though a few respondents expressed doubts about strips’ effectiveness in reducing overdose harms. Some SSPs are expanding education and naloxone training/distribution in the broader community, beyond PWUD and their friends/family. Respondents described several ongoing barriers to preventing overdose death, including not reaching certain groups at risk of overdose (PWUD who do not inject, PWUD experiencing homelessness, and PWUD of color), an inconsistent naloxone supply and lack of access to intranasal naloxone in particular, inadequate funding, underestimates of overdoses, legal/policy barriers, and community stigma.

**Conclusions:**

SSPs remain essential in preventing overdose deaths amid record numbers likely driven by increased fentanyl use and COVID-19-related impacts. These findings can inform efforts to support SSPs in this work. In the face of ongoing barriers, support for SSPs—including increased resources, political support, and community partnership—is urgently needed to address the worsening overdose crisis.

## Background

Drug overdose is a worsening crisis in the US, where overdose is a leading cause of accidental death and an important contributor to declining life expectancy. [1] US overdose deaths reached a record high and continued to surge through 2021. [2, 3] The majority (64%) of overdose deaths during May 2020–April 2021 involved synthetic opioids—primarily non-pharmaceutical fentanyl and fentanyl analogs—and over half of these deaths involved additional substances, most commonly stimulants (methamphetamine or cocaine). [4] Longitudinal interrupted time series studies have identified increases in overdose-related emergency department visits concurrent with the onset of the coronavirus (COVID-19) pandemic in some states [5, 6].

Syringe services programs (SSPs) were established to prevent transmission of HIV and other blood-borne pathogens among people who use drugs (PWUD) through the distribution of safe injection equipment. [7, 8] Now, these programs provide a range of vital services, including services to prevent overdose death such as distribution of naloxone (overdose reversal medication) and overdose prevention education. [9] SSPs play a critical role in addressing the overdose crisis, serving a population at high risk for overdose who may be unlikely to access healthcare in other settings due to stigma and other barriers[10–12].

Recent qualitative studies have reported SSPs’ perspectives on how the COVID-19 pandemic impacted their operations. [13–15] In-depth information regarding how SSP programs are responding to the current surge in overdoses and what barriers they are facing to these efforts is needed to inform the allocation of resources and formulation of policies to support SSPs in preventing overdose death. We conducted semi-structured qualitative interviews with a geographically diverse sample of US SSP leadership and staff to understand: (1) perspectives on factors contributing to the overdose surge, (2) SSPs’ current strategies to respond to the overdose surge, and (3) ongoing barriers to SSPs’ efforts to prevent overdose death.

## Methods

### Study sample and recruitment

The study sample was recruited from an existing cohort of US SSPs that participated in a prior qualitative study; the original cohort was drawn from the North American Syringe Exchange Network directory, and sampling and recruitment of this cohort have been previously described. [13] SSPs that participated in the prior study (*N* = 31) were invited to participate in this study; programs were first contacted by email and received up to two follow-up contacts by email and/or telephone. Any SSP leadership or staff (including volunteers) were eligible to participate on behalf of their organization; we conducted one interview per program. Here, we refer to people who participated in this study as “respondents” to avoid confusion with SSP participants (a common term referring to people who receive SSP services). The University of Washington Human Subjects Division determined that this study was not human subjects research.

### Data collection

Interviews were conducted remotely from 2/11/2021 to 4/23/2021 by four interviewers who were knowledgeable about SSP services and drug overdose (MAC, NDF, EH, and AMJ). The semi-structured interview guide included questions about respondents’ perception of trends in overdose and contributing factors, their organization’s current approach to preventing overdose death among SSP participants, and barriers they face to addressing overdose (additional topics were addressed in the interview; these data are being analyzed separately). Respondents also provided information about their program in a brief online questionnaire, including current distribution model and approaches, the number of SSP sites operated by their organization, and the estimated number of syringes distributed annually by their organization. Organization location and type (i.e., health department-operated or nonprofit/community-based) were collected from respondents in an earlier round of data collection. [13] Respondents received a $50 electronic gift card as compensation for their time and effort.

### Data analysis

Interviews were audio-recorded and transcribed. One interview was conducted in Spanish and transcribed into English. Interview data were analyzed using a Rapid Assessment Process (RAP), [16] which allows for rapid analysis of qualitative data in time-sensitive contexts. [17–19] RAP methods involve summarizing findings and key quotations from each transcript under each code, rather than coding transcripts line-by-line. These methods have been found to produce similar findings to traditional qualitative coding methods, [19] and a RAP approach was chosen to allow for faster dissemination of actionable results in the context of the ongoing pandemic and overdose crisis. Each interview transcript was summarized using a template in which responses and illustrative quotations were organized under codes. We took a primarily deductive approach to content analysis (i.e., codes were primarily developed a priori rather than developed from the data). [20] A priori codes were developed based on the content of the interview guide, and emergent codes were added during analysis. The template contained a section for each code with definitions and examples, with space to add a bulleted summary and relevant quotations from the transcript that fit under each code. The template thus functioned as a codebook guiding analyses and was updated as needed to capture emergent codes throughout the analysis process by consensus of the analytic team.

Transcripts were summarized using the template by four analysts with experience in qualitative analysis and substance use research (EJA, ESB, MAC, and MCF). The summaries were iteratively reviewed by these analysts and a qualitative lead with expertise in qualitative research and RAP methods (ECW), who helped resolve discrepancies as needed. At first each transcript was independently summarized by two analysts (*n* = 14); once clear consensus on themes was reached, the remaining transcripts (*n* = 13) were each summarized by one analyst. The final transcript summaries were combined into a single matrix document displaying data within each code across each interview. The matrix was used to develop a final summary of themes, which was reviewed by the broader study team and was sent to respondents to check accuracy.

## Results

Twenty-seven respondents completed interviews, reflecting 27 unique programs. SSP characteristics are described in Table 1. Most SSPs were in urban areas and were nonprofit or community-based organizations rather than health department-operated. Fixed sites (i.e., providing services at a single, fixed location), mobile delivery (i.e., providing services from a mobile location, such as a van), and secondary exchange (i.e., having SSP participants distribute to and return supplies from other PWUD) were all common approaches to distributing harm reduction supplies. Thirteen programs operated only one site, eight operated 2–3, three operated 4–9, and three operated 10 or more. Programs’ estimated number of syringes distributed annually (which could include multiple sites) ranged from 10,000 to 5,800,000.

**Table 1:** Characteristics of SSPs that participated in qualitative interviews, 2021 (N = 27)

	*n*	%
*Geographic region*		
Northeast Midwest South West US Territories	8 9 5 4 1	30% 33% 19% 15% 4%
*Urbanicity/rurality*		
Urban Rural	20 7	74% 26%
*SSP type*		
Health department-operated Nonprofit/community-based organization	4 23	15% 85%
*Current syringe distribution model*^*a*^		
One-for-one Needs-based Other	2 16 9	7% 59% 33%
*Current distribution approach(es)*^b^		
Fixed site Mobile delivery Secondary exchange Other	20 21 18 4	74% 78% 67% 15%
*Number of sites operated* 1 2–3 4–9 10 +	13 8 3 3	48% 30% 11% 11%
	Median	Range
Estimated syringes distributed annually^c^	300,000	10,000–5,800,000

^a^“One-for-one” refers to distributing one syringe for every syringe returned; “needs-based” refers to distributing syringes without restrictions/requirements related to returning used syringes

^b^SSPs could report using more than one distribution approach

^c^Missing one program that was unable to respond to this question

### Perspectives on factors contributing to the overdose surge

Respondents generally reported that overdoses were substantially increasing, though some said this trend did not apply to their area or that local available data were insufficient to be informative. A few perceived that overdoses were increasing more rapidly among PWUD of color compared to white PWUD. Respondents discussed multiple factors that they believed were contributing to the overdose surge.

#### Increase in presence and use of fentanyl and other potent substances

Respondents reported that a large increase in fentanyl use was a major driver of the overdose surge, including both intentional and unintentional use of fentanyl on its own or when present in other substances. Not all respondents mentioned fentanyl use, but there were not clear patterns across location or urbanicity/rurality in perception of fentanyl use.

“*We are in the worst overdose crisis that [location] has ever seen…in the last couple of years, fentanyl has really been here…our fentanyl overdoses have doubled for 2020*.” (Respondent 18, urban, community-based organization)

One respondent also described their suspicion that increased presence of xylazine (a tranquilizer) in the drug supply was contributing to overdose.

“*I think it’s a supply/contamination issue…We’ve seen what we are suspecting is xylazine as a cut down here, recently. Which is a tranquilizer. So, we’ve heard…that naloxone wasn’t as effective as this person thought it would be…We don’t have testing kits for it, but that’s what we suspect is going on*.” (Respondent 19, urban, community-based organization)

One respondent felt that increased presence of fentanyl in non-opioid substances, combined with lack of outreach in communities where opioid use has been less common, was an important driver of overdose increases among PWUD of color.

“*…overdoses for Black and Brown [PWUD] had raised up, which we definitely think is attributable to fentanyl and the fact that fentanyl's being found in drugs of choice for folks that don’t use opiates…the outreach has not been there in those neighborhoods…there's a lot of folks that didn’t feel like [naloxone] was applicable to them because they didn’t use opiates*.” (Respondent 15, urban, community-based organization)

A couple of respondents had observed reduced heroin availability leading to more fentanyl and methamphetamine use among participants. They speculated that changes in drug availability might be related to the pandemic.

“*I don’t know for sure… more and more people are using fentanyl, because less and less real heroin is on the streets. So, I think that that probably is directly affected by COVID, with all the different lock-downs in movement*.” (Respondent 10, urban, community-based organization)

#### Impact of the COVID-19 pandemic on drug use and access to resources

Respondents reported that overdoses were increasing due to an increase in solitary drug use resulting from isolation created by COVID-19-related social distancing practices.

“*We have noticed an increase in…overdose deaths because many participants are trying to be on their own because of the COVID issues, which causes them to inject on their own, and if they overdose there is no one to respond*.” (Respondent 20, urban, community-based organization)

Respondents also reported that the pandemic was leading to abrupt increases in drug use or return to use after a period of not using, resulting in increased overdose risk. They cited life disruptions (e.g., changes in access to money, resources such as support groups) and declining mental health as pandemic-related reasons for changes in drug use.

“…*many [participants] injected a little [when it was difficult to get money], but when they managed to get money, they injected the amount they were injecting before, and then they were more susceptible to overdose. This is how several participants died*.” (Respondent 20, urban, community-based organization)

“*I do know several folks that whatever was working for them, whatever kind of meetings or support they were getting, COVID really put a little hitch in that…A lot of people returning to use after maybe having considered themselves in recovery for a time*.” (Respondent 24, urban, community-based organization)

*“…this pandemic has been scary for a lot of people...Folks are probably feeling a little more anxiety and are using to cope with that*.” (Respondent 26, urban, community-based organization)

One respondent had noticed an increase in suicidality among participants and felt that some of the increase in overdose deaths was driven by intentional as well as unintentional overdoses.

“*We saw an increase in suicidality among our participants, really feeling completely alone and left behind…some of these overdoses are intentional…we don't want to minimize the extent to which unintentional overdose is driving the overdose epidemic, but with COVID, mental health has just collapsed*.” (Respondent 27, urban, community-based organization)

### SSPs’ current strategies to respond to the overdose surge

Respondents described multiple strategies currently being used by their programs to prevent overdose and overdose death among participants.

#### Increasing naloxone distribution

Most SSPs had substantially increased naloxone distribution in response to rising overdose rates. This increase included expanding distribution to SSP participants who use only non-opioid drugs due to increased likelihood of these drugs containing fentanyl. It also included distributing more doses per person, and respondents noted that multiple doses may be needed to reverse a single overdose involving fentanyl or another strong drug (e.g., “purple heroin”).

“*We've been definitely trying to make sure that everybody [who] comes in has naloxone, is aware that we have it and can give it to them*.” (Respondent 8, urban, health department-operated)

“*I’ve asked for two doses in each bag instead of one because of the fentanyl*.” (Respondent 21, rural, community-based organization)

Some respondents had specifically increased distribution of the intranasal formulation of naloxone because they had found it easier to use than the injectable for some participants and their family and friends.

*“[Intranasal naloxone] just seems to work best for our community. Especially for family members that may need to use it*.” (Respondent 16, rural, community-based organization)

Respondents described several facilitators to increasing naloxone distribution. Some SSPs had received increased funding for naloxone, or naloxone donated by other organizations (e.g., other SSPs, health department). Logistical facilitators included the US Food and Drug Administration authorizing the extension of naloxone’s expiration date and greater flexibility in storage requirements. [21].

“*…the one-year expiration date from when they may get [naloxone], they expanded it to a year beyond that, so that’s helped. And the temperature that you have to store it has also changed so you have more flexibility with that. So, those have been the things that have helped people feel they can use it better*.” (Respondent 21, rural, community-based organization)

Respondents also described multiple strategies to increase the reach of naloxone distribution. Some were encouraging secondary distribution, in which SSP participants are given extra naloxone to give to other PWUD in their social networks.

“*We’re giving out 6-10 kits per person, in part because we’re messaging to them, ‘Hey, do you have folks in your network that you can take and give naloxone kits to?*’” (Respondent 7, urban, health department-operated)

Some SSPs were providing education and naloxone to other organizations (e.g., social service agencies, jails) so that those organizations could distribute it.

“*We also have one of our staff members that works with…social service agencies, in terms of doing naloxone education and leaving naloxone for them to have available*.” (Respondent 16, rural, community-based organization)

#### Adapting overdose education

Several respondents described having adapted overdose education efforts to address the evolving crisis. This included increasing awareness of fentanyl’s high overdose risk and the high prevalence of fentanyl in the drug supply.

“*We’re really working to [create] more awareness about overdoses, the risks of fentanyl use. Or at least, the risk of fentanyl use if you don’t know that there’s fentanyl in your dope*.” (Respondent 26, urban, community-based organization)

They also described approaching participants who use only non-opioid drugs, in addition to those who use opioids, to educate them about how to prevent overdose death from fentanyl that may be in the drugs they are using.

“*We have a very high stimulant use population in this state…and we know everything is adulterated with fentanyl as well, or other analogs. So, we've been coaching folks that, regardless of what your drug of choice is, make sure you have [naloxone] on hand*.” (Respondent 14, urban, community-based organization)

One respondent also described how they often needed to address stigma related to fentanyl use and overdose among people who do not intentionally use fentanyl or opioids:

“*There's a lot of meth users and some cocaine that were just like, ‘Oh, well, I don’t have to worry about that. I don’t touch that stuff.'…just letting people know…‘I know you're not seeking this out.’ Because they may have overcome some heroin use in the past, but just letting them know that it's in there, and to be aware of that, and it's not anything they’ve done wrong. So, just trying to reduce the stigma, because there's a lot, when it comes to fentanyl and fentanyl users*.” (Respondent 1, urban, health department-operated)

#### Distributing fentanyl test strips

Several SSPs were distributing fentanyl test strips to participants so they could test their drugs for fentanyl. Some felt that these were effective in changing behavior to reduce overdose risk.

“*90 percent of folks are doing something different when [the fentanyl test] comes up positive. They’re using less, they’re pushing the plunger more slowly, they’re telling friends, they’re using [naloxone], or some people are throwing away the drug entirely*.” (Respondent 18, urban, community-based organization)

However, some respondents had doubts about the effectiveness of fentanyl test strips, either because they were concerned about false negatives or thought that participants who would most benefit from them (i.e., people without substantial opioid tolerance) do not typically use them.

“*We don’t give the things that test for [fentanyl] and all the strips, because the studies on them aren’t great…a lot of times it would say there isn’t and there is…I just thought it was a waste of funding to do that, but I might be wrong*.” (Respondent 17, rural, community-based organization)

*“…there’s a heck of a lot of people…that have a tolerance for fentanyl for whom the fentanyl test strip is really losing its value…It may help a new person who’s just initiating or has been using other kinds of substances…People that are overdosing on fentanyl that aren’t tolerant to opioids at all…they’re usually not testing for it, honestly, unfortunately*.” (Respondent 24, urban, community-based organization)

#### Engaging the broader community

Several respondents described how their organizations had taken action to engage members of the broader community (i.e., beyond SSP participants and their friends/family). These efforts included distributing naloxone and providing naloxone training to pharmacy students at the local university, medical professionals, and general community members, as well as distributing overdose education materials (e.g., pamphlets) at local agencies and businesses (e.g., gas stations, motels).

“*Last summer we had a community Naloxone giveaway with education and giving out Naloxone in five of our six counties that we serve*.” (Respondent 16, rural, community-based organization)

One respondent described how in community trainings they communicate that everyone in the community has a role to play in preventing overdose death, even if they are uncomfortable administering naloxone.

“*I do this in the trainings that I do with community members*…*Everybody doesn’t have to be the one to administer it. But if you’re willing to be out there, carry naloxone. Have it available wherever you are, and tell other people…how to use it…making it welcoming to everybody regardless of what type of role they want to play in it*.” (Respondent 7, urban, health department-operated)

### Barriers to preventing overdose death

Respondents also described several ongoing barriers to preventing overdose death faced by their organizations.

#### Not reaching everyone at risk of overdose

Some respondents expressed concern that their SSPs were not sufficiently reaching certain groups at risk of overdose, including PWUD who don’t inject, PWUD experiencing homelessness who have been displaced, and PWUD of color.

“*And the big scary piece that we’ve been talking about lately is all the folks that we’re starting to hear who have switched from heroin to pills…if they’re not coming to us because they’re not injecting anymore, we’re not reaching them*.” (Respondent 7, urban, health department-operated)

“…*the [police department] and homeless services made a really concerted effort to break up all the encampments…it was where a big chunk of our participants were sleeping, and we knew where they were…So, that's been really hard on everybody. It's caused overdose deaths. It's meant that we're engaging less people because we don't know where they are*.” (Respondent 27, urban, community-based organization)

One respondent discussed how their program did not yet adequately serve PWUD of color, and their hope that starting a mobile unit would increase access in these communities.

*“I think the latest report…shows about 40 percent of the overdose deaths were either Black or Brown people. We probably have less than five percent of Black and Brown people actually accessing our program. Our program right now is overwhelmingly White…I think being able to see our mobile unit out in the community will right that wrong…if you see me out in the community and this is what we're doing and nobody's getting arrested or that sort of thing, then that might change some perspectives and people might be more willing to access it*.” (Respondent 12, urban, community-based organization)

#### Inconsistent supply of available naloxone and/or inadequate funding

Some respondents noted that naloxone is expensive, and their programs needed more funding to keep up with increased distribution. Many observed that the supply of available naloxone was often inconsistent even with adequate funding in place.

“*It’s been challenging to get naloxone sometimes. I don’t know if that’s supply shortage or sourcing…we could use a steady supply*.” (Respondent 25, urban, community-based organization)

Several respondents specifically mentioned unavailability of intranasal naloxone. Some felt this was needed to adequately serve their community.

“*I really need nasal [naloxone]…it’s Indian Country, and there are so many Natives’ grandmothers taking care of their kids, and taking care of their grandkids, and eyesight issues, they might have arthritis, but trying to load a syringe is hard…there should be greater availability of nasal [naloxone]*.” (Respondent 22, urban, community-based organization)

One respondent described how the assumption that people who inject drugs should be comfortable using injectable naloxone can be a barrier to SSPs obtaining intranasal naloxone.

“*…people who may not be in the drug community may just assume, ‘Well, if they’re willing to [inject drugs], then they can do anything. So, why are you telling me that they need to use nasal naloxone as opposed to intramuscular naloxone?’*…*a needle to use the intramuscular naloxone is a much thicker needle…People may not have started drugs like, ‘I’m going to use needles.’… they might be still be needle-phobic…if that means that they’d rather take the nasal over the intramuscular, then I really want to have the nasal available to give them*.” (Respondent 11, rural, community-based organization)

#### Perception that overdoses are undercounted

Several respondents perceived that nonfatal and/or fatal overdoses were being undercounted in their area. They suspected this was due to overdoses being successfully reversed with naloxone without any involvement from emergency responders or law enforcement, and to overdose deaths being misclassified.

“*We are seeing a lot of people who are performing…successful reversals, and we’re keeping track of those types of numbers. But those things wouldn’t get reported because they’re not calling in to the police or the hospitals*.” (Respondent 11, rural, community-based organization)

One respondent suggested that underestimation of overdoses negatively impacts SSPs when naloxone allocations are decreased.

“*[The state supplies] naloxone to us. But then, they only allocated X amount, not understanding the trend of overdoses…if we didn’t have naloxone out there, the overdose rate would be even higher than it is*.” (Respondent 13, urban, community-based organization)

#### Legal/policy barriers and community stigma

Some respondents described how local laws or policies impeded prevention of overdose death. One respondent reported that their location did not have a Good Samaritan law that provided legal protection for people who intervene in an overdose, and SSP services other than naloxone distribution (e.g., distributing syringes) were considered illegal in another respondent’s location which made it difficult to provide services.

“*…many people in the community are afraid to administer naloxone because…there is a Good Samaritan Act, but it doesn’t cover people who intervene in an overdose. So, many people do not want to intervene in an overdose because they are afraid that the law doesn’t protect them*.” (Respondent 20, urban, community-based organization)

Additionally, several respondents pointed out the need for safer consumption facilities to adequately prevent overdose death, and some were involved in efforts to pass legislation that would allow these to be established.

“*I think that a lot of issues that we're talking about would be a lot lower if there were spaces, particularly in areas with high overdose and overdose death rates, had places for folks to go and use safely with supervision*.” (Respondent 15, urban, community-based organization)

Some respondents also described how stigma in the community, especially lack of support from other agencies such as law enforcement, medical facilities, and public health departments, impeded prevention of overdose death.

“*Because we're a public health department in a rural, conservative county, I think there's not a lot of freedom in how we can reach our population…I don’t have the ability to have a texting service…people have asked me if we have our own Facebook page, but our health commissioner's not for that. And I know that our police are really against…[naloxone]-ing people. Not all of them, but one of the things that we have to do when we call 911 is say, ‘Someone's not breathing,’ instead of, ‘We think it's a drug overdose*.’” (Respondent 1, urban, health department-operated)

## Discussion

In this qualitative study examining perspectives of SSP leadership and staff, respondents perceived that overdose deaths were increasing overall and particularly among PWUD of color and reported that the surge is being driven by a large increase in fentanyl use and COVID-19-related factors, as well as increased presence of xylazine. Their perspectives aligned with quantitative data showing an ongoing increase in overdose deaths, [3] that overdose deaths are increasing more rapidly among Black and American Indian/Alaska Native PWUD than White PWUD, [22] that most overdose deaths now involve fentanyl and many involve multiple substances, [4, 23] and that xylazine is increasingly involved in overdose deaths. [24] Perspectives also aligned with studies examining PWUD perspectives finding that—for some—the COVID-19 pandemic has led to increased mental health issues, using drugs alone more frequently, and changes in financial resources impacting drug use. [25–28] Respondents also described using multiple strategies and facing ongoing barriers to preventing overdose death. Some of the strategies and barriers described in this study aligned with qualitative studies of SSPs conducted prior to or early in the pandemic, such as inadequate funding, policy barriers, and community stigma. [14, 29, 30] Some findings from the present study are novel, such as SSPs’ specific need for intranasal naloxone and barriers to obtaining it. Our finding that stigma may act as a barrier to addressing fentanyl-related overdose risk among some PWUD who only intend to use non-opioid drugs is also novel.

Findings from the present study may have implications for agencies and individuals who support the work of SSPs, including health departments, funders, policymakers, community leaders, and technical assistance providers. More consistent funding for SSPs and a steady naloxone supply may be needed to support increased naloxone distribution. Respondents perceived that naloxone was difficult to obtain, which is likely the result of a widespread naloxone shortage driven by the pandemic and supply chain disruptions. [31] Researchers and harm reduction leaders have made urgent calls for federal policymakers to address this problem, including increasing production, lowering costs, and making naloxone available over the counter. [31, 32] Some respondents noted that their SSPs relied on naloxone donated from other organizations, and harm reduction leaders have called for increased flexibility to move naloxone between organizations (e.g., healthcare facilities, police departments, and SSPs). [31] Increasing SSPs’ access to intranasal naloxone will likely require increased funding for SSPs to buy a more expensive formulation, and/or efforts to lower the cost of this formulation. [31] It may also require correcting the erroneous assumption that people who inject drugs never need or prefer intranasal naloxone, as well as raising awareness of the importance of providing intranasal naloxone to family and friends of PWUD through SSPs. One respondent perceived an increase in suicidality among participants. Rates of suicidality among SSP participants should be assessed to determine if SSPs should consider implementing mental health screening and linkage to services.

Respondents varied in their perceptions of fentanyl test strips—some felt they reduced overdose risk, while others felt they were not effective due to either the potential for false negatives or lack of uptake among participants who would most benefit (i.e., people without substantial opioid tolerance). Fentanyl test strips have been shown to be effective at detecting fentanyl, though high concentrations of stimulants and cutting agents may result in false positives. [33] Studies suggest that fentanyl test strips can promote safer drug use behaviors and are considered acceptable/useful by PWUD. [34–38] However, PWUD have also described barriers to using testing strips, such as concern about experiencing withdrawal if they have to delay use. [39] More robust evidence on the strips’ accuracy when used by PWUD to test their drugs prior to consumption is needed, [40, 41] and this should be shared with SSPs. More research is needed assessing how PWUD respond to positive or negative fentanyl test strip results to inform and optimize messaging to PWUD about their use. It may be beneficial for SSPs to communicate with each other regarding how they have effectively implemented use of fentanyl test strips within the context of other overdose prevention services.

SSPs may need support in increasing mobile service delivery and community outreach, which may include additional funding and support in establishing new partnerships. These efforts could help SSPs expand naloxone distribution and possibly reach more people at risk of overdose who are currently less likely to access SSPs, such as PWUD who do not inject, PWUD experiencing homelessness, and PWUD of color. [42, 43] These efforts may also help SSPs reduce stigma and foster support for harm reduction services in the community through education and increasing awareness of the positive impact of harm reduction. [30]

Some respondents perceived that overdoses were undercounted in their area, a concern that has been reported previously, [30] and further noted that underestimation of nonfatal overdoses due to naloxone reversals not being captured in surveillance data could lead to misguided reductions in supplies. There is a need for more widespread and standardized reporting of both fatal and nonfatal overdoses, and integrating overdose reversal data tracked by SSPs with data from emergency departments and other sources may increase accuracy of nonfatal overdose counts. Additionally, state/local leadership and funders should be aware that overdoses might be undercounted and work directly with SSPs to meet their resource needs. Local laws/policies that support SSPs and overdose reversal are needed, as there is considerable variation across the US in the authorization of SSPs as well as whether Good Samaritan laws explicitly protect people who request help in the event of overdose from arrest. [44, 45] Finally, several respondents called for safer consumption facilities to address the worsening overdose crisis, such as the overdose prevention centers recently established in New York City [46].

This study has limitations. First, perspectives of SSP leadership and staff may or may not align with actual trends in drug use/overdose and participants’ experiences. Findings should be considered in the context of epidemiological studies and research involving SSP participants. However, it is important to understand the perspectives of SSP leadership and staff as they directly inform what SSPs prioritize and how they approach this issue. Second, while qualitative research is important for identifying and describing a range of perspectives and for allowing SSP leadership and staff to describe their experiences in their own words, findings cannot be considered representative and provide limited insight into prevalence and broader patterns. Future quantitative research informed by these qualitative findings (e.g., surveys distributed to all SSPs) can assess the overall prevalence of different strategies and barriers to preventing overdose death among SSPs, as well as variation across factors such as organizational characteristics, geographic region, and urban/rural location. This research may help policymakers, funders, and other leaders prioritize strategies to support SSPs. For example, it may be that SSPs in different regions are differentially impacted by the naloxone shortage, or that SSPs serving specific communities report higher need for intranasal naloxone.

## Conclusions

Qualitative interviews were conducted with 27 US SSP leadership and staff. Respondents perceived that the current overdose surge was being driven primarily by a large increase in fentanyl use on its own and when present in other substances, as well as COVID-19-related factors including increased solitary drug use, life disruptions, and worsened mental health among PWUD. SSPs are responding to this surge with multiple strategies including increasing naloxone distribution, distributing fentanyl test strips, adapting overdose education, and engaging the broader community in naloxone distribution. Ongoing barriers to preventing overdose death faced by SSPs include not reaching certain groups at risk of overdose, an inconsistent supply of naloxone and lack of access to intranasal naloxone in particular, inadequate funding, nonfatal and/or fatal overdoses being undercounted in their area, legal/policy barriers, and community stigma. These findings can inform efforts to support SSPs in preventing overdose death. In the face of ongoing barriers, support for SSPs—including increased resources, political support, and community partnership—is urgently needed to address the worsening overdose crisis.

## Data Availability

Data are not publicly available due to protect participant privacy.

## Abbreviations

COVID-19: Coronavirus
PWUD: People who use drugs
SSP: Syringe services program
RAP: Rapid assessment process
US: United States
